# Update: COVID-19 Among Workers in Meat and Poultry Processing Facilities ― United States, April–May 2020

**DOI:** 10.15585/mmwr.mm6927e2

**Published:** 2020-07-10

**Authors:** Michelle A. Waltenburg, Tristan Victoroff, Charles E. Rose, Marilee Butterfield, Rachel H. Jervis, Kristen M. Fedak, Julie A. Gabel, Amanda Feldpausch, Eileen M. Dunne, Connie Austin, Farah S. Ahmed, Sheri Tubach, Charles Rhea, Anna Krueger, David A. Crum, Johanna Vostok, Michael J. Moore, George Turabelidze, Derry Stover, Matthew Donahue, Karen Edge, Bernadette Gutierrez, Kelly E. Kline, Nichole Martz, James C. Rajotte, Ernest Julian, Abdoulaye Diedhiou, Rachel Radcliffe, Joshua L. Clayton, Dustin Ortbahn, Jason Cummins, Bree Barbeau, Julia Murphy, Brandy Darby, Nicholas R. Graff, Tia K. H. Dostal, Ian W. Pray, Courtney Tillman, Michelle M. Dittrich, Gail Burns-Grant, Sooji Lee, Alisa Spieckerman, Kashif Iqbal, Sean M. Griffing, Alicia Lawson, Hugh M. Mainzer, Andreea E. Bealle, Erika Edding, Kathryn E. Arnold, Tomas Rodriguez, Sarah Merkle, Kristen Pettrone, Karen Schlanger, Kristin LaBar, Kate Hendricks, Arielle Lasry, Vikram Krishnasamy, Henry T. Walke, Dale A. Rose, Margaret A. Honein, COVID-19 Response Team, COVID-19 Response Team, Keith Amoroso, Yvette Diallo, Kathie Fazekas,, Phillip J. Finley, Jennifer Fuld, Jodie L. Guest, Jocelyn J. Herstein, Erin D. Kennedy, James V. Lawler, John J. Lowe, Alexander Neifert, Michelle M. Schwedhelm, Nebraska Medicine, Jonathan M. Steinberg, Douglas B. Trout, Max Zarate-Bermudez

**Affiliations:** ^1^CDC COVID-19 Emergency Response; ^2^Epidemic Intelligence Service, CDC; ^3^Arizona Department of Health Services; ^4^Colorado Department of Public Health and Environment; ^5^Georgia Department of Public Health; ^6^Idaho Department of Health and Welfare; ^7^Illinois Department of Public Health; ^8^Kansas Department of Health and Environment; ^9^Kentucky Department for Public Health; ^10^Maine Center for Disease Control and Prevention; ^11^Maryland Department of Health; ^12^Massachusetts Department of Public Health; ^13^Missouri Department of Health and Senior Services; ^14^Nebraska Department of Health and Human Services; ^15^New Mexico Department of Health; ^16^Pennslyvania Department of Health; ^17^Pennsylvania Department of Agriculture; ^18^Rhode Island Department of Health; ^19^South Carolina Department of Health and Environmental Control; ^20^South Dakota Department of Health; ^21^Tennessee Department of Health; ^22^Utah Department of Health; ^23^Virginia Department of Health; ^24^Washington State Department of Health; ^25^Wisconsin Department of Health Services; ^26^Wyoming Department of Health.; Rhode Island Department of Health; CDC; CDC; CDC; CDC; Emory University, Atlanta, Georgia; Global Center for Health Security; University of Nebraska Medical Center; CDC; Global Center for Health Security; University of Nebraska Medical Center; Global Center for Health Security; University of Nebraska Medical Center; Rhode Island Department of Health; Global Center for Health Security; South Dakota Department of Health; CDC; CDC.

*On July 7, 2020, this report was posted online as an *MMWR *Early Release.*

Meat and poultry processing facilities face distinctive challenges in the control of infectious diseases, including coronavirus disease 2019 (COVID-19) (*1*). COVID-19 outbreaks among meat and poultry processing facility workers can rapidly affect large numbers of persons. Assessment of COVID-19 cases among workers in 115 meat and poultry processing facilities through April 27, 2020, documented 4,913 cases and 20 deaths reported by 19 states (*1*). This report provides updated aggregate data from states regarding the number of meat and poultry processing facilities affected by COVID-19, the number and demographic characteristics of affected workers, and the number of COVID-19–associated deaths among workers, as well as descriptions of interventions and prevention efforts at these facilities. Aggregate data on confirmed COVID-19 cases and deaths among workers identified and reported through May 31, 2020, were obtained from 239 affected facilities (those with a laboratory-confirmed COVID-19 case in one or more workers) in 23 states.* COVID-19 was confirmed in 16,233 workers, including 86 COVID-19–related deaths. Among 14 states reporting the total number of workers in affected meat and poultry processing facilities (112,616), COVID-19 was diagnosed in 9.1% of workers. Among 9,919 (61%) cases in 21 states with reported race/ethnicity, 87% occurred among racial and ethnic minority workers. Commonly reported interventions and prevention efforts at facilities included implementing worker temperature or symptom screening and COVID-19 education, mandating face coverings, adding hand hygiene stations, and adding physical barriers between workers. Targeted workplace interventions and prevention efforts that are appropriately tailored to the groups most affected by COVID-19 are critical to reducing both COVID-19–associated occupational risk and health disparities among vulnerable populations. Implementation of these interventions and prevention efforts^†^ across meat and poultry processing facilities nationally could help protect workers in this critical infrastructure industry.

Distinctive factors that increase meat and poultry processing workers’ risk for exposure to SARS-CoV-2, the virus that causes COVID-19, include prolonged close workplace contact with coworkers (within 6 feet for ≥15 minutes) for long time periods (8–12 hour shifts), shared work spaces, shared transportation to and from the workplace, congregate housing, and frequent community contact with fellow workers. Many of these factors might also contribute to ongoing community transmission (*1*). To better understand the effect of COVID-19 on workers in these facilities nationwide, on June 6, 2020, CDC requested that state health departments report aggregate surveillance data through May 31, 2020, for workers in all meat and poultry processing facilities affected by COVID-19, including 1) the number and type of such facilities that had reported at least one confirmed COVID-19 case among workers, 2) the total number of workers in affected facilities, 3) the number of workers with laboratory-confirmed COVID-19, and 4) the number of COVID-19–related worker deaths. States reported COVID-19 cases determined by the Council of State and Territorial Epidemiologists confirmed case definition.^§^ States were asked to report demographic characteristics and symptom status of workers with COVID-19. Testing strategies and methods for collecting symptom data varied by workplace. Proportional distributions for demographic characteristics and symptom status were calculated for cases among workers in 21 states after excluding missing and unknown values; data were missing for sex in 25% of reports, age in 24%, race/ethnicity in 39%, and symptom status in 37%. States also provided information (from direct observation or from management at affected facilities) regarding specified interventions and prevention efforts that were implemented. A random-effects logistic regression model was used to obtain an estimate of the pooled proportion of asymptomatic (SARS-CoV-2 detected but symptoms never develop) or presymptomatic (SARS-CoV-2 detected before symptom onset) infections at the time of testing among workers who had positive SARS-CoV-2 test results. Five states provided prevalence data from facility-wide testing of 5,572 workers in seven facilities. Modeling was conducted and 95% confidence intervals (CIs) were calculated, with facilities treated as the random effect, using SAS software (version 9.4; SAS Institute).

Twenty-eight (56%) of 50 states responded, including 23 (82%) that reported at least one confirmed COVID-19 case among meat and poultry processing workers. Overall, 239 facilities reported 16,233 COVID-19 cases and 86 COVID-19–related deaths among workers (Table 1). The median number of affected facilities per state was seven (interquartile range = 3–14). Among 14 states reporting the total number of workers in affected facilities, 9.1% of 112,616 workers received diagnoses of COVID-19. The percentage of workers with COVID-19 ranged from 3.1% to 24.5% per facility.

**TABLE 1:** Laboratory-confirmed COVID-19 cases among workers in meat and poultry facilities — 23 states, April–May 2020*

State	Type of meat/poultry in affected facilities	No. (%)			
		Facilities affected	Workers in affected facilities^†^	Confirmed COVID-19 cases among workers	COVID-19–related deaths^§^
Arizona	Beef	1	1,750	162 (9.3)	0 (0)
Colorado	Beef, bison, lamb, poultry	7	7,711	422 (5.5)	9 (2.1)
Georgia	Poultry	14	16,500	509 (3.1)	1 (0.2)
Idaho	Beef	2	797	72 (9.0)	0 (0)
Illinois	Beef, pork, poultry	26	N/A	1,029 (―)	10 (1.0)
Kansas	Beef, pork, poultry	10	N/A	2,670 (―)	8 (0.3)
Kentucky	Pork, poultry	7	7,633	559 (7.3)	4 (0.7)
Maine	Poultry	1	411	50 (12.2)	1 (2.0)
Maryland	Poultry	2	2,036	208 (10.2)	5 (2.4)
Massachusetts	Poultry, other	33	N/A	263 (―)	0 (0)
Missouri	Beef, pork, poultry	9	8,469	745 (8.8)	2 (0.3)
Nebraska	Beef, pork, poultry	23	26,134	3,438 (13.2)	14 (0.4)
New Mexico	Beef, pork, poultry	2	550	24 (4.4)	0 (0)
Pennsylvania	Beef, pork, poultry, other	30	15,548	1,169 (7.5)	8 (0.7)
Rhode Island	Beef, pork, poultry, other	6	N/A	78 (―)	0 (0)
South Carolina	Beef, pork, poultry, other	16	N/A	97 (―)	0 (0)
South Dakota	Beef, pork, poultry	4	6,500	1,593 (24.5)	3 (0.2)
Tennessee	Pork, poultry, other	7	N/A	640 (―)	2 (0.3)
Utah	Beef, pork, poultry	4	N/A	67 (―)	1 (1.5)
Virginia	Pork, poultry, other	14	N/A	1,109 (―)	10 (0.9)
Washington	Beef, poultry	7	4,452	468 (10.5)	4 (0.9)
Wisconsin	Beef, pork, veal	14	14,125	860 (6.1)	4 (0.5)
Wyoming	Beef	0	N/A	1 (―)	0 (0)
**Total^¶^**	**Beef, bison, lamb, pork, poultry, veal, other**	**239**	**112,616**	**16,233**	**86**
Combined total**	**―**	**264**	**―**	**17,358**	**91**

**Abbreviations:** COVID-19 = coronavirus disease 2019; N/A = not available.

* Data reported through May 31, 2020. Five states that responded to the data request did not report any laboratory-confirmed COVID-19 cases among workers in the animal slaughtering and processing industry; 22 states with animal slaughtering and processing facilities did not respond to the data request. The 13 states that contributed to both an earlier assessment and this update provided any updates to previously reported data, in addition to reporting new cases and facilities, through May 31, 2020.

^†^ Among 14 of 23 states reporting the number of workers in affected workplaces, 9.1% of workers received diagnoses of COVID-19.

^§^ Percentage of deaths among cases.

^¶^ Data on workers with COVID-19 from 23 states that submitted data to this report.

** Combining data on workers with COVID-19 (1,125), COVID-19–related deaths (five), and COVID-19–affected facilities (25) through April 27 from six states that contributed to an earlier assessment of COVID-19 among meat and poultry processing workers that did not submit updated data to this report (https://www.cdc.gov/mmwr/volumes/69/wr/mm6918e3.htm?s_cid=mm6918e3_w).

Twenty-one states provided information on demographic characteristics and symptom status of workers with COVID-19. Among the 12,100 (75%) and 12,365 (76%) patients with information on sex and age, 7,288 (60%) cases occurred among males, and 5,741 (46%) were aged 40–59 years, respectively (Figure). Among the 9,919 (61%) cases with race/ethnicity reported, 5,584 (56%) were in Hispanics, 1,842 (19%) in non-Hispanic blacks (blacks), 1,332 (13%) in non-Hispanic whites (whites), and 1,161 (12%) in Asians. Symptom status was reported for 10,284 (63%) cases; among these, 9,072 (88%) workers were symptomatic, and 1,212 (12%) were asymptomatic or presymptomatic.

**FIGURE:**
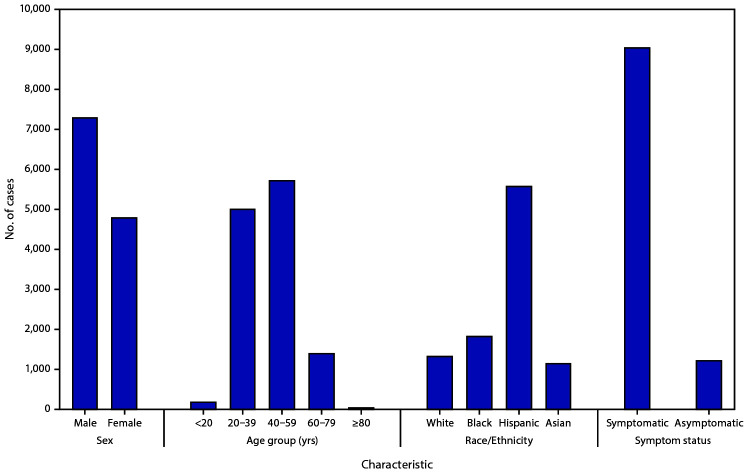
Characteristics^*^^,^^†^ of reported laboratory-confirmed COVID-19 cases among workers in meat and poultry processing facilities — 21 states, April–May 2020^§^ **Abbreviation:** COVID-19 = coronavirus disease 2019. * The analytic dataset excludes cases reported by states that were missing information on sex (4,133), age (3,868), race/ethnicity (6,314), and symptom status (5,949). White, black, and Asian workers were non-Hispanic; Hispanic workers could be of any race. ^†^ Testing strategies and methods for collecting symptom data varied by workplace. Symptom status was available for a single timepoint, at the time of testing or at the time of interview. ^§^ Data reported through May 31, 2020.

Among 239 facilities reporting cases, information on interventions and prevention efforts was available for 111 (46%) facilities from 14 states. Overall, 89 (80%) facilities reported screening workers on entry, 86 (77%) required all workers to wear face coverings, 72 (65%) increased the availability of hand hygiene stations, 70 (63%) educated workers on community spread, and 69 (62%) installed physical barriers between workers (Table 2). Forty-one (37%) of 111 facilities offered testing for SARS-CoV-2 to workers; 24 (22%) reported closing temporarily as an intervention measure.

**TABLE 2:** Interventions and prevention efforts implemented by facilities in response to COVID-19 among workers in 111 meat and poultry processing facilities* —14 states, April–May 2020^†^

Intervention/Prevention effort	COVID-19–affected facilities, no. (%^§^)		
	Implemented intervention	Did not implement intervention	Intervention status unknown
Worker screening on entry	89 (80)	5 (5)	17 (15)
Required universal face covering	86 (77)	5 (5)	20 (18)
Added hand hygiene stations	72 (65)	8 (7)	31 (28)
Educated employees on community spread	70 (63)	13 (12)	28 (25)
Installed physical barriers between workers	69 (62)	17 (15)	25 (23)
Staggered shifts	57 (51)	17 (15)	37 (33)
Offered SARS-CoV-2 testing to employees¶	41 (37)	35 (32)	35 (32)
Removed financial incentives (e.g., attendance bonuses)	33 (30)	20 (18)	58 (52)
Closed facility temporarily	24 (22)	69 (62)	18 (16)
Reduced rate of animal processing	23 (21)	14 (12)	74 (67)
Decreased crowding of transportation to worksite	17 (15)	10 (9)	84 (76)

**Abbreviation:** COVID-19 = coronavirus disease 2019.

* Affected facilities defined as those having one or more laboratory-confirmed COVID-19 cases among workers.

^†^ Based on data collected through May 31, 2020.

^§^ Because of rounding, row percentages might not equal 100%.

^¶^ Testing strategies varied by facility.

Among seven facilities that implemented facility-wide testing, the crude prevalence of asymptomatic or presymptomatic infections among 5,572 workers who had positive SARS-CoV-2 test results was 14.4%. The pooled prevalence estimated from the model for the proportion of asymptomatic or presymptomatic infections among workers in meat and poultry processing facilities was 11.2% (95% CI = 0.9%–23.1%).

## Discussion

The animal slaughtering and processing industry employs an estimated 525,000 workers in approximately 3,500 facilities nationwide (*2*,*3*). Combining data on workers with COVID-19 and COVID-19–related deaths identified and reported through May 31 from 23 states (16,233 cases; 86 deaths) with data from an earlier assessment through April 27 (1,125 cases; five deaths) (*1*) that included data from six states that did not contribute updated data to this report,^¶^ at least 17,358 cases and 91 COVID-19–related deaths have occurred among U.S. meat and poultry processing workers.

The effects of COVID-19 on racial and ethnic minority groups are not yet fully understood; however, current data indicate a disproportionate burden of illness and death among these populations (*4*,*5*). Among animal slaughtering and processing workers from the 21 states included in this report whose race/ethnicity were known, approximately 39% were white, 30% were Hispanic, 25% were black, and 6% were Asian.** However, among 9,919 workers with COVID-19 with race/ethnicity reported, approximately 56% were Hispanic, 19% were black, 13% were white, and 12% were Asian, suggesting that Hispanic and Asian workers might be disproportionately affected by COVID-19 in this workplace setting. Ongoing efforts to reduce incidence and better understand the effects of COVID-19 on the health of racial and ethnic minorities are important to ensure that workplace-specific prevention strategies and intervention messages are tailored to those groups most affected by COVID-19.

The proportion of asymptomatic or presymptomatic SARS-CoV-2 infections identified in investigations of COVID-19 outbreaks in other high-density settings has ranged from 19% to 88% (*6*,*7*). Among cases in workers with known symptom status in this report, 12% of patients were asymptomatic or presymptomatic; however, not all facilities performed facility-wide testing, during which these infections are more likely to be identified. Consequently, many asymptomatic and presymptomatic infections in the overall workforce might have gone unrecognized, and the approximations for disease prevalence in this report might underestimate SARS-CoV-2 infections. Recently derived estimates of the total proportion of asymptomatic and presymptomatic infections from data on COVID-19 investigations among cruise ship passengers and evacuees from Wuhan, China, ranged from 17.9% to 30.8%, respectively (*8*,*9*). The estimated proportion of asymptomatic and presymptomatic infections among meat and poultry processing workers (11.2%) is lower than are previously reported estimates and should be reevaluated as more comprehensive facility-wide testing data are reported.

In coordination with state and local health agencies, many meat and poultry processing facilities have implemented interventions to reduce transmission or prevent ongoing exposure within the workplace, including offering testing to workers.^††^ Expanding interventions across these facilities nationwide might help protect workers in this industry. Recognizing the interaction of workplace and community, many facilities have also educated workers about strategies for reducing transmission of COVID-19 outside the workplace.^§§^

The findings in this report are subject to at least seven limitations. First, only 28 of 50 states responded; 23 states with COVID-19 cases among meat and poultry processing facility workers submitted data for this report. In addition, only facilities with at least one laboratory-confirmed case of COVID-19 among workers were included. Thus, these results might not be representative of all U.S. meat and poultry processing facilities and workers. Second, delays in identifying workplace outbreaks and linking cases or deaths to outbreaks might have resulted in an underestimation of the number of affected facilities and cases among workers. Third, data were not reported on variations in testing availability and practices, which might influence the number of cases reported. Fourth, industry data were used for race/ethnicity comparisons; demographic characteristics of total worker populations in affected facilities were not available, limiting the ability to quantify the degree to which some racial and ethnic minority groups might be disproportionately affected by COVID-19 in this industry. Reported frequencies of demographic and symptom data likely underestimate the actual prevalence because of missing data, which limits the conclusions that can be drawn from descriptive analyses. Fifth, information on interventions and prevention efforts was available for a subset of affected facilities and therefore might not be generalizable to all facilities. Information was subject to self-report by facility management, and all available intervention efforts might not have been captured. Further evaluation of the extent of control measures and timing of implementations is needed to assess effectiveness of control measures. Sixth, symptom data collected at facility-wide testing was self-reported and might have been influenced by the presence of employers. Finally, workers in this industry are members of their local communities, and their source of exposure and infection could not be determined; for those living in communities experiencing widespread transmission, exposure might have occurred within the surrounding community as well as at the worksite.

High population-density workplace settings such as meat and poultry processing facilities present ongoing challenges to preventing and reducing the risk for SARS-CoV-2 transmission. Collaborative implementation of interventions and prevention efforts, which might include comprehensive testing strategies, could help reduce COVID-19–associated occupational risk. Targeted, workplace-specific prevention strategies are critical to reducing COVID-19–associated health disparities among vulnerable populations Lessons learned from investigating outbreaks of COVID-19 in meat and poultry processing facilities could inform investigations in other food production and agriculture workplaces to help prevent and reduce COVID-19 transmission among all workers in these essential industries.

**Summary**

**What is already known about this topic?**

COVID-19 outbreaks among meat and poultry processing facility workers can rapidly affect large numbers of persons.

**What is added by this report?**

Among 23 states reporting COVID-19 outbreaks in meat and poultry processing facilities, 16,233 cases in 239 facilities occurred, including 86 (0.5%) COVID-19–related deaths. Among cases with race/ethnicity reported, 87% occurred among racial or ethnic minorities. Commonly implemented interventions included worker screening, source control measures (universal face coverings), engineering controls (physical barriers), and infection prevention measures (additional hand hygiene stations).

**What are the implications for public health practice?**

Targeted workplace interventions and prevention efforts that are appropriately tailored to the groups most affected by COVID-19 are critical to reducing both COVID-19–associated occupational risk and health disparities among vulnerable populations.

## Footnotes

* Arizona, Colorado, Georgia, Idaho, Illinois, Kansas, Kentucky, Maine, Maryland, Massachusetts, Missouri, Nebraska, New Mexico, Pennsylvania, Rhode Island, South Carolina, South Dakota, Tennessee, Utah, Virginia, Washington, Wisconsin, and Wyoming.

† https://www.cdc.gov/coronavirus/2019-ncov/community/organizations/meat-poultry-processing-workers-employers.html.

§ https://wwwn.cdc.gov/nndss/conditions/coronavirus-disease-2019-covid-19/case-definition/2020/.

¶ Delaware, Iowa, Mississippi, North Carolina, Ohio, and Texas did not contribute data to this report.

** Data produced for 21 of 23 states (Colorado and Kansas did not provide information on demographic characteristics and symptom status of cases) using the Bureau of Census American Community Survey (CMS) Public Use Microdata Sample (PUMS) query tool (https://www.census.gov/programs-surveys/acs/data/pums.html). Employment summaries were based on the American Community Survey 2014–2018 5-year PUMS estimates. Workforce estimates for Bureau of Census Industry Code 1180 (Animal Slaughtering and Processing) were tabulated by race/ethnicity using recoded detailed Hispanic origin and race.

†† https://www.cdc.gov/coronavirus/2019-ncov/community/worker-safety-support/hd-testing.html.

§§ https://www.cdc.gov/coronavirus/2019-ncov/community/community-mitigation.html.
